# What can we learn from other countries—lessons from the CAJC Happiness Report

**DOI:** 10.1515/iss-2018-0032

**Published:** 2019-04-05

**Authors:** Anjali A. Roeth

**Affiliations:** Department of General, Visceral and Transplant Surgery, University Hospital RWTH Aachen, Pauwelsstr. 30, 52074 Aachen, Germany; Department of Surgery, Maastricht University, Maastricht, The Netherlands; Surgical Working Group of “Young Surgeons” (CAJC) of the German Society for General and Visceral Surgery (DGAV), Berlin, Germany,

**Keywords:** different countries, job satisfaction, surgical residency, worldwide

## Abstract

Most countries have their own programs and requirements for surgical residency. To investigate the differences as well as the advantages and disadvantages of the programs and to explore the happiness of the residents in the different countries, the Surgical Working Group of “Young Surgeons” of the German Society for General and Visceral Surgery has designed a questionnaire. It focuses on three parts: structural and legal requirements, operating room (OR)- and non-OR-related content of the program, and contentment of the residents. In this opinion paper, first the results are shared by the description of the programs in nine different countries. It is shown that the requirements to become a surgeon differ highly between the different countries. Nonetheless, a structured curriculum, the possibility of feedback or a surgical mentoring program, and transparency regarding the OR schedule seem to be important to all residents to reach job satisfaction.

## Introduction

The residency training program in the United States has been introduced by the famous surgeon William Stewart Halsted more than 100 years ago and has not changed much since those days [1]. He called it “residency” program as the doctors actually lived in the hospital. Before, there was just an apprentice-master relationship between the surgeon and his student. Today, most countries have their own programs for surgical residency, which differ a lot. To explore the differences as well as the advantages and disadvantages of the different residency programs, the Surgical Working Group of “Young Surgeons” (CAJC) of the German Society for General and Visceral Surgery designed a questionnaire based on a national questionnaire that has been already published by our work group [2]. The purpose of the study is to describe a “World Happiness Report for Residents” similar to the “Word Happiness Report” of the United Nations [3]. The study is still ongoing, but here some early results are being highlighted to point out how different surgical residency can be and which aspects may be used to improve residency. As the information is based on the results of the opinion survey, there is no claim to completeness.

## Questionnaire

The questionnaire contains 32 questions and consists of three main parts:

1. After four introductory questions, nine questions are on structural and legal requirements such as qualifications needed, duration, final examination, and existence of a structured curriculum;
2. Six questions are regarding the practical content of the residency, i.e. at what time which procedure is being performed and if the substeps of a procedure may be done by the resident; and
3. The last part addresses the non-operating room (OR)-related training opportunities such as simulation programs, theoretical knowledge, or surgical mentoring programs and consists of nine questions.

The questionnaire also tests the contentment of the residents and contains a comment section to address which aspects they particularly like and dislike about their program.

In the following, some of the different national programs are described in alphabetical order of the countries (only if there are two or more replies to the questionnaire). At the end of each description, the key aspects and contentment are described as well as the lessons learned so far. Note that these are only descriptive, early results used for this opinion paper as the study is still ongoing and will then be published with the final results.

### Brazil

In Brazil, the residency to become a general surgeon lasts only 2 years. The residency is only possible in large hospitals and ends with an oral exam. After this time, most physicians leave to practice as surgeons in smaller hospitals or private practices. No additional examination is needed. Only very few surgeons continue their training in large hospitals to specialize even more, e.g. in hepatopancreatobiliary (HPB) surgery or upper gastrointestinal (GI) surgery.

*Key aspects/contentment of residents (early results):* Residents seem to like that they only learn the basic skills they will need later within 2 years.

*What can we learn:* Focusing on surgical procedures with respect to skills needed later is useful.

### Germany

In Germany, every hospital can train surgical residents. In the beginning, 2 years of a so-called common trunk are required, which includes 6 months at an emergency department and 6 months in an intensive care unit. After this, the more specialized part of residency follows, ending with an oral examination conducted by the state medical association (Landesaerztekammer). During the second part, residents can specialize in general, visceral, vascular, thoracic, heart, plastic, trauma, or pediatric surgery. Every 6 months, the resident gets a feedback talk with the surgeon in charge of surgical education. Whether or not this takes place is largely dependent on each individual hospital. Nonetheless, for the application for the oral examination at the end of residency, it needs to be proven that both the resident and the surgeon in charge have documented the feedback talks at least once a year (§8 WBO, e.g. [4]).

*Key aspects/contentment of residents (early results):* Residents seem to like that they can subspecialize after the first 2 years of broader surgical education. Contentment seems to rise with regularly performed feedback talks.

*What can we learn:* Feedback talks on a regular basis help the residents.

### Great Britain

Great Britain has a very structured and centrally organized residency program. For the first 2 years, one serves as house officer as part of a consultant team. There are different rotations, each with internal further training possibilities at least once a week. After these so-called “foundation years”, there are 2–4 years as senior house officer. During this time, a catalogue of surgeries has to be performed. The time as senior house officer ends with a big clinical and practical exam (MRCS) at the Royal Colleges of Surgeons. To become a consultant, 6 years of training as specialist registrar have to be fulfilled thereafter, which consist of 3 basic years, 1–2 years of out-of-program experience, and 2 years of subspecialization. Every year, a record-in-training assessment takes place, where the consultant gives structured feedback to the trainee and vice versa. Apart from surgical and clinical skills, communicational and scientific skills also are addressed. Due to this very structured program, residency is mainly possible in NHS hospitals that undertake surgery. Despite the very structured program, there can be discrepancy between the time residents are allowed to work according to the European Working Time Directive and the time needed for patient safety.

*Key aspects/contentment of residents (early results):* Residents seem to like the very structured program and grade their happiness very highly. Also, the training of soft skills is considered very important and useful.

*What can we learn:* Structured residency programs that are transparent and not only focus on OR skills are highly appreciated.

### Greece

In Greece, after receiving a diploma at the university, one has to apply for a residency at a specific hospital. As the waiting lists are long, it takes up to 3 years to get a slot in general surgery. The quality of the residency seems to be dependent on the favor of the superiors. Nonetheless, there is a logbook with certain numbers of procedures that are required for the oral examination at the end of the residency. Residents see a lot space for improvement of the Greek system, especially regarding the salary, as they often earn less than 1000 € per month.

*Key aspects/contentment of residents (early results):* Long waiting lists for getting a slot, low income, and nontransparent assignment of surgical procedures seem to lead to dissatisfaction.

*What can we learn:* Transparent assignment of surgical procedures and salary that appreciates the work done are important.

### Italy

To get a residency slot in Italy, one has to pass a written entrance examination. Also, an enrollment fee has to be paid. The residency in general surgery lasts 6 years. Weeks are very structured with 1 day of teaching, 2 days of working on the ward and the outpatient clinic, and 2 days in the operation theater. An exam has to be passed every year, and at the end of the residency, a scientific paper has to be written.

*Key aspects/contentment of residents (early results):* Weeks are very structured, leading to job satisfaction as the residents often know their schedule several months in advance.

*What can we learn:* Structured work schedules several months in advance make it easier to also plan work as well as leisure time.

### The Netherlands

In The Netherlands, the teaching hospital decides who gets a residency slot. As places are scarce, the graduates from medical school often take a position as Ph.D. student or ANIOS (physician working mainly on the ward without being in an official residency and without getting the chance to train to become a surgeon). Thereby, they get to know the staff better and hence have better chances of being successful in the interview to get a residency slot. Residency for surgery lasts 6 years, but often the residents specialize already in the last 2 years (e.g. HPB surgery or colorectal surgery). In the Dutch system, the residents get constant feedback, especially regarding their surgical skills. For each procedure performed, an Objective Structured Assessment of Technical Skills is filled out by the supervisor with positive and negative feedback as well as an evaluation of the competence level at which the procedure was performed (i.e. independently versus only under supervision, etc.).

*Key aspects/contentment of residents (early results):* Residents seem to be very happy with their program once they get in. Especially, the rather early subspecialization as well as the feedback on all procedures performed are much appreciated.

*What can we learn:* With constant feedback, the residents get to know their strengths and weaknesses and can improve accordingly.

### Republic of Singapore

In Singapore, residency can only be done at the university hospitals. Besides the clinical training, this involves structured education as well as research opportunities that the residents are encouraged to do. The program is very structured with an exam at the end of each year. During the first 3 years, the junior residents learn a broad basis of general surgery and surrounding disciplines such as gynecology, plastic surgery, and otorhinolaryngology. During the following 2 years as a senior resident, there is the opportunity to develop a subspecialization besides having more responsibilities in the core general surgery rotations with a mentored surgical management of the patients together with the junior resident and the consultant.

*Key aspects/contentment of residents (early results):* The structured program and the mentorship are very much appreciated. Residents seem to also like the very broad education they receive during as junior residents.

*What can we learn:* A mentorship lasting for some time helps the residents to improve their skills.

### Sweden

After getting the medical degree at the university, Swedish physicians must fulfill an internship of 18–21 months including 3–6 months of surgery, 3–6 months of internal medicine, 3 months of psychiatry, and 6 months of general practice. The internship ends with an exam to get the medical license and is followed by 5 years of surgical residency. As each hospital is responsible for the training, smaller hospitals often do not have the sufficient number of cases to train residents. Each resident is assigned to a personal instructor with a special education. There are assigned study times of 4 h per week to gain theoretical knowledge. In addition, mandatory courses have to be taken, which are organized by the Swedish Surgical Society. These include basic skills, advanced trauma life support, hernia repair, endocrine surgery, breast surgery, and upper and lower GI surgery.

*Key aspects/contentment of residents (early results):* Residents seem to dislike the extremely broad basis they get during internship. The surgical residency itself leads to contentment as there is a mentorship with personal instructor and transparent, mandatory training courses.

*What can we learn:* Centrally organized training courses can help make the surgical education more transparent.

### United States

The residency programs in the United States are very structured. To get into a residency program, medical students have to apply for the programs, which is followed by interviews at the different hospitals. Students and residency programs then submit a “rank order-list” to a centralized matching service. On “Match Day” in March, all students at the different medical schools learn where they are going to start their residency. After 1 year of internship, 4 years of surgical residency follow. Most programs have a very structured schedule with dedicated educational time during which also morbidity and mortality conferences or Grand Rounds have to be attended. Operative procedures are often recorded in an online database by the Accreditation Council for Graduate Medical Education. During surgical residency, the work hours are often long and used to average between 88- and 95-h workweeks depending on the program size [5]. In recent years, the limit has been set to 80-h workweeks. At the end of each year, the American Board of Surgery in Training Examination has to be taken. If the residents do not fulfill the requirements, they have to repeat the year. After residency, often a fellowship is taken to subspecialize further.

*Key aspects/contentment of residents (early results):* Although the structured programs with dedicated educational time are valued, job satisfaction suffers due to the long workweeks.

*What can we learn:* A well-adjusted work-life balance is important for contentment.

## Discussion and conclusion

These early results underline that the requirements to become a surgeon differ highly between the different countries. Although all graduates from the multiple residency programs can call themselves “surgeons”, they are only partly comparable. Nonetheless, there seem to be patterns that result in higher contentment of the residents. Key aspects are a structured curriculum with guidance on which procedure should be done at which time. Furthermore, the possibility of feedback from the superior to improve the skills, in particular with the existence of a surgical mentoring program, is very much appreciated by the residents. In addition, transparency regarding the OR schedule, i.e. which resident is allowed to do which procedure, seems to be important to the residents of all countries. Some of these aspects have already been addressed by the CAJC before as a result of our national survey and workshops [2], [6]. Also, von Websky et al. have already investigated with a Global Job Satisfaction Instrument, a tool known mainly from the industry and large companies, the job satisfaction of residents in Germany, the United Kingdom, and Switzerland. They found that the factors that affected the satisfaction most are the assignment of surgery procedures according to skills, availability of a structured training curriculum, a good working climate among residents, and the option for part-time work [7]. In contrast, as our study investigates the contentment in different countries, we will be able to find out not only what the residents would like to have but also if others, who are actually working with the above-mentioned conditions (e.g. a very structured curriculum), are really happy, depending on the respective country. Nonetheless, the above-mentioned early results have the limitation that they are taken from a questionnaire, i.e. they might be influenced by the perception of the resident filling it out. Therefore, a larger number of residents from each country have to be asked to make it representative. In addition, we have to rely on the answers, and we did not investigate if, for example, a structured curriculum is also used not only in theory but also in everyday life.

Although the length and conditions of surgical training vary substantially between different countries, lessons can be learned from all of the different programs. The possibility of transferring these to residency programs in other countries is of course dependent on the respective background and structure. As the study on differences in residencies around the world is still ongoing, we welcome anyone who wants to participate.

## Supplementary Material

The article (iss-2018-0032) offers reviewer assessments as supplementary material.
