# Supplementary material for: What can we learn from other countries—lessons from the CAJC Happiness Report
Source: Innov Surg Sci. 2019 Apr 5;4(1):25–9. doi: 10.1515/iss-2018-0032 (PMC6754059; doi:10.1515/iss-2018-0032)
Supplement: Supplementary file 1 [file iss-4-20180032-s001.pdf]

Reviewer Assessment

Anjali A. Roeth*

What can we learn from other countries—lessons from the CAJC Happiness Report

https://doi.org/10.1515/iss-2018-0032
Received September 24, 2018; accepted March 15, 2019

***Corresponding author: Dr. med. Dipl.-Phys. Anjali A. Roeth,** M.D., M.Sc., Department of General, Visceral and Transplant Surgery, University Hospital RWTH Aachen, Pauwelsstr. 30, 52074 Aachen, Germany; Department of Surgery, Maastricht University, Maastricht, The Netherlands; and Surgical Working Group of "Young Surgeons" (CAJC) of the German Society for General and Visceral Surgery (DGAV), Berlin, Germany, E-mail: aroeth@ukaachen.de

Reviewers' Comments to Original Submission

Reviewer 1: Timm Franzke

Oct 03, 2018

**Reviewer Recommendation Term:**	Accept with minor revision
**Overall Reviewer Manuscript Rating:**	N/A
**Custom Review Questions**	**Response**
Is the subject area appropriate for you?	3
Does the title clearly reflect the paper's content?	2
Does the abstract clearly reflect the paper's content?	2
Do the keywords clearly reflect the paper's content?	3
Does the introduction present the problem clearly?	4
Are the results/conclusions justified?	2
How comprehensive and up-to-date is the subject matter presented?	3
How adequate is the data presentation?	1 - Low/No
Are units and terminology used correctly?	4
Is the number of cases adequate?	4
Are the experimental methods/clinical studies adequate?	2
Is the length appropriate in relation to the content?	3
Does the reader get new insights from the article?	2
Please rate the practical significance.	2
Please rate the accuracy of methods.	2
Please rate the statistical evaluation and quality control.	2
Please rate the appropriateness of the figures and tables.	3
Please rate the appropriateness of the references.	4
Please evaluate the writing style and use of language.	3
Please judge the overall scientific quality of the manuscript.	2

Open Access. © 2019 Roeth A.A., published by De Gruyter. This work is licensed under the Creative Commons Attribution 4.0 Public License.

Are you willing to review the revision of this manuscript?	Yes

Comments to Authors:
Grundsätzlich verstehe und begrüße ich den Ansatz die chirurgische Ausbildung weltweit zu vergleichen und so eine gewisse Transparenz herzustellen. Auch die Frage nach dem „Glücklichsein" wir im Rahmen des Generationswechsel sicherlich interessant (Thema: Nachwuchsmangel/ja ein Anliegen der DGAV). Es handelt sich hier um die ersten Ergebnisse eine Befragung!?! Von dem Anspruch einen happiness-report zu erstellen, ist jedoch noch nicht viel zu sehen, respektive zu lesen. Auch die Frage, was wir lernen können, bleibt unbeantwortete. Mir ist bewusst, dass es sich um erste Ergebnisse handelt, dann muss dies jedoch auch betont werden. Die Vision, das Ziel sollte jedoch wenigstens benannt werden/erkennbar sein. Ebenso sollte ein Ausblick auf weitere Veröffentlichungen gegeben werden. Weiterhin bleibt unklar, wie viele Kollegen wurden befragt? Wieviel haben geantwortet? Ebenso wichtig, wie das Vorhandensein einer strukturierten Ausbildung auf dem Papier, ist jedoch auch die Umsetzung der Vorgaben. Wird dies auch untersucht?

Reviewer 2: John Primrose

Nov 30, 2018

**Reviewer Recommendation Term:**	Accept with minor revision
**Overall Reviewer Manuscript Rating:**	60

**Custom Review Questions**	**Response**
Is the subject area appropriate for you?	5 - High/Yes
Does the title clearly reflect the paper's content?	5 - High/Yes
Does the abstract clearly reflect the paper's content?	5 - High/Yes
Do the keywords clearly reflect the paper's content?	5 - High/Yes
Does the introduction present the problem clearly?	4
Are the results/conclusions justified?	3
How comprehensive and up-to-date is the subject matter presented?	5 - High/Yes
How adequate is the data presentation?	4
Are units and terminology used correctly?	N/A
Is the number of cases adequate?	N/A
Are the experimental methods/clinical studies adequate?	N/A
Is the length appropriate in relation to the content?	4
Does the reader get new insights from the article?	5 - High/Yes
Please rate the practical significance.	5 - High/Yes
Please rate the accuracy of methods.	N/A
Please rate the statistical evaluation and quality control.	N/A
Please rate the appropriateness of the figures and tables.	N/A
Please rate the appropriateness of the references.	5 - High/Yes
Please evaluate the writing style and use of language.	4
Please judge the overall scientific quality of the manuscript.	3
Are you willing to review the revision of this manuscript?	Yes

Comments to Authors:
This manuscript is from a German surgical trainees group and is describing surgical training schemes in a number of countries. It is work in progress rather than the finished article and I have reviewed it as such. I am uncertain what led to the selection of particular countries. I cannot say how accurate the descriptions are save for the UK. In the UK training can take place in most NHS hospitals which undertake surgery, and most have trainees. Independent sector hospitals may or may have trainees attending with a supervisor but it is correct that they will not be based there. In the UK hours of work is meant to comply with EWTD and rotas are intended to be compliant and observed. The conflict when trainees are required to leave because of time restraints but need to stay for patients safety is a regular problem that detracts from job satisfaction. It is worth noting that in many specialised areas of surgery a post training scheme or "fellowship" is usually needed eg HPB surgery. Note is made of the point that the length of training varies considerably in different systems and thus the competency of the end product is not at all compatible. In terms of language the paper is satisfactory. The word "slot" should replace "spot".

Authors' Response to Reviewer Comments

ANSWERS TO EDITORIAL COMMENTS

Dear Authors,
thank you for submitting this interesting article on the different training situations of residents all over the world. It provides a good general overview of the different concepts.
Overall I think that this article is of great interest to the readers of ISS. Some minor improvements could be done to provide some more details on the actual "happiness" in the respective countries and to present the "pearls and pitfalls" of the different systems, so that the reader can have an immediate take away upon reading the article.

General Ideas that would improve the article:

1) A short Materials and Methods section describing the questionnaire (even though it has already been published) would be helpful.

ANSWER: Thank you for these very good suggestions. A paragraph describing the questionnaire more closely has been added to the manuscript.

2) After the description of every country I would add two short points describing "the most important takeaway" in one sentence and "what could be improved on" in one sentence (although the last point is no must..).

ANSWER: After each description of a country, two points were added: 1. "Key aspects/ contentment of residents (early results)" and 2. "What can we learn". I agree that these help summarize the most important aspects and lessons learned.

3) If this data is available (seeing as these are preliminary results) a short statement on the "happiness" of the surgeons in the respective country would be nice; maybe even in the form of a map highlighting the countries investigated. Especially since you refer to the contentment as part of the discussion it should be mentioned during the results.

ANSWER: The contentment of the residents in the different countries was descriptively added to the two points after each country (see Answer 2). With this, is becomes clearer, which parts of the programs are liked or disliked by the residents. As the number of residents interviewed is not yet representative for every country, often only the tendency was being described and it was pointed out that these are only early results.

ANSWERS TO REVIEWERS COMMENTS

REVIEWER #1
Grundsätzlich verstehe und begrüße ich den Ansatz die chirurgische Ausbildung weltweit zu vergleichen und so eine gewisse Transparenz herzustellen. Auch die Frage nach dem „Glücklichsein" wir im Rahmen des Generationswechsel sicherlich interessant (Thema: Nachwuchsmangel/ja ein Anliegen der DGAV). Es handelt sich hier um die ersten Ergebnisse eine Befragung!?! Von dem Anspruch einen happiness-report zu erstellen, ist jedoch noch nicht viel zu sehen, respektive zu lesen. Auch die Frage, was wir lernen können, bleibt unbeantwortete. Mir ist bewusst, dass es sich um erste Ergebnisse handelt, dann muss dies jedoch auch betont werden. Die Vision, das Ziel sollte jedoch wenigstens benannt werden/erkennbar sein. Ebenso sollte ein Ausblick auf weitere Veröffentlichungen gegeben werden. Weiterhin bleibt unklar, wie viele Kollegen wurden befragt? Wieviel haben geantwortet? Ebenso wichtig, wie das Vorhandensein einer strukturierten Ausbildung auf dem Papier, ist jedoch auch die Umsetzung der Vorgaben. Wird dies auch untersucht?

ANSWER: Vielen Dank für diese wirklich hilfreichen Anmerkungen, die ich alle im überarbeiteten Manuskript addressiert habe. Es handelt sich dabei um folgende Veränderungen:
As described above, each description of a country's residency program is now followed by two aspects to summarize the description: 1. "Key aspects/ contentment of residents (early results)" and 2. "What can we learn". With this, the question what we can learned is answered. In addition, I have underlined several times throughout the manuscript that these are just very early results of an ongoing survey which will be published in the future, as suggested. Only countries where already two or more residents have answered the questionnaire where included. This is now expressed in the new paragraph on the questionnaire itself. Thank you for pointing out that there might be a difference in theory and everyday life. I have addressed this for Germany (regarding the regularity of feedback talks) and also added a part with limitations if this opinion paper in the discussion.

REVIEWER #2
This manuscript is from a German surgical trainees group and is describing surgical training schemes in a number of countries. It is work in progress rather than the finished article and I have reviewed it as such. I am uncertain what led to the selection of particular countries. I cannot say how accurate the descriptions are save for the UK. In the UK training can take place in most NHS hospitals which undertake surgery, and most have trainees. Independent sector hospitals may or may have trainees attending with a supervisor but it is correct that they will not be based there. In the UK hours of work is meant to comply with EWTD and rotas are intended to be compliant and observed. The conflict when trainees are required to leave because of time restraints but need to stay for patients safety is a regular problem that detracts from job satisfaction. It is worth noting that in many specialised areas of surgery a post training scheme or “fellowship” is usually needed eg HPB surgery. Note is made of the point that the length of training varies considerably in different systems and thus the competency of the end product is not at all compatible. In terms of language the paper is satisfactory. The word “slot” should replace “spot”.

ANSWER: Thank you for these valuable comments. I have addressed them also in the revised manuscript. I have added a paragraph on the questionnaire itself, in which the selection of these 9 countries is explained. Thank you also for the further information on residency in the UK, I have changed and completed the part on the UK accordingly. Also, I have stressed in the paper, that the article is an opinion paper with very early results and that the results from the study itself will be published after receiving a sufficient and representative amount of questionnaires. The word “residency spot” was replaced by “residency slot” throughout the paper.

Reviewers’ Comments to Revision

Reviewer 1: Timm Franzke

Mar 15, 2019

**Reviewer Recommendation Term:**	Accept
**Overall Reviewer Manuscript Rating:**	N/A
**Custom Review Questions**	**Response**
Is the subject area appropriate for you?	3
Does the title clearly reflect the paper’s content?	3
Does the abstract clearly reflect the paper’s content?	3
Do the keywords clearly reflect the paper’s content?	4
Does the introduction present the problem clearly?	4
Are the results/conclusions justified?	2
How comprehensive and up-to-date is the subject matter presented?	4
How adequate is the data presentation?	2
Are units and terminology used correctly?	4
Is the number of cases adequate?	1 - Low/No
Are the experimental methods/clinical studies adequate?	3
Is the length appropriate in relation to the content?	3
Does the reader get new insights from the article?	4
Please rate the practical significance.	4
Please rate the accuracy of methods.	2
Please rate the statistical evaluation and quality control.	2
Please rate the appropriateness of the figures and tables.	2
Please rate the appropriateness of the references.	2
Please evaluate the writing style and use of language.	4
Please judge the overall scientific quality of the manuscript.	3
Are you willing to review the revision of this manuscript?	Yes

Comments to Authors:
No further comments.

Reviewer 2: John Primrose

Feb 19, 2019

**Reviewer Recommendation Term:**	Accept with minor revisions
**Overall Reviewer Manuscript Rating:**	66
**Custom Review Questions**	**Response**
Is the subject area appropriate for you?	3
Does the title clearly reflect the paper's content?	4
Does the abstract clearly reflect the paper's content?	4
Do the keywords clearly reflect the paper's content?	4
Does the introduction present the problem clearly?	4
Are the results/conclusions justified?	4
How comprehensive and up-to-date is the subject matter presented?	3
How adequate is the data presentation?	4
Are units and terminology used correctly?	4
Is the number of cases adequate?	N/A
Are the experimental methods/clinical studies adequate?	N/A
Is the length appropriate in relation to the content?	3
Does the reader get new insights from the article?	3
Please rate the practical significance.	3
Please rate the accuracy of methods.	3
Please rate the statistical evaluation and quality control.	N/A
Please rate the appropriateness of the figures and tables.	N/A
Please rate the appropriateness of the references.	4
Please evaluate the writing style and use of language.	4
Please judge the overall scientific quality of the manuscript.	3
Are you willing to review the revision of this manuscript?	Yes

Comments to Authors:
No further comments.
